# The effectiveness of cervical mucus electrical impedance compared to basal body temperature to determine fertility window

**DOI:** 10.1186/s40834-024-00276-w

**Published:** 2024-05-06

**Authors:** Suzanne Tabbaa, Sealy Hambright, Katie J. Sikes, Gary Levy, Jan Rydfors

**Affiliations:** 1 Lady Technologies, San Francisco, CA USA; 2 Colorado State University, Fort Collins, CO USA; 3 Fertility Cloud, Palo Alto, CA USA; 4 Stanford University Medical Center, Stanford, CA USA; 5 Freyja Medical Clinic, Redwood City, CA USA; 6 Orthopaedic Surgery, UCSF School of Medicine, 1001 Potrero Ave, #346, 94110, San Francisco, CA USA

**Keywords:** Fertility window, Ovulation function, Electrical impedance

## Abstract

**Background:**

Serial serum hormone measurements and transvaginal ultrasound are reliable measures to predict ovulation. These measures are inconvenient and expensive therefore, basal body temperature charting (BBT) and urine ovulation predictor kits (OPK) for luteinizing hormone are often used to determine the 6-day fertile window. However, BBT does not clearly change until 1–2 days after ovulation. Additionally, while OPK can indicate positivity prior to ovulation, false readings are common. A novel alternative approach involves measuring electrolyte trends in cervical mucus using electrical impedance spectroscopy. Cervical mucus electrolyte measurements are associated with hormone level changes during the menstrual cycle. The purpose of this study was to compare the effectiveness of cervical mucus electrical impedance and basal body temperature. We sought to determine if cervical mucus electrolyte measurements provided improved detection of the ovulation day and therefore, improve fertility timing for women.

**Methods:**

14 healthy women between 18 and 44 years of age with normal menstrual cycles were enrolled in the Observational Study. Participants measured BBT and cervical mucus electrical impedance daily for 3 menstrual cycles using Kegg (Lady Technologies Inc. San Francisco, California, USA). Ovulation date for each cycle was confirmed by measuring hormone levels in urine and serum, and by vaginal ultrasound.

**Results:**

Electrical impedance was significantly different between the follicular phase versus ovulatory date (*p* = 0.007) and between the luteal phase versus the ovulatory date (*p* = 0.007). A significant difference in the rate of change of cervical impedance measurements in the pre-ovulatory follicular phase was found compared to BBT (*p* = 0.0225). The sensitivity (+ 7.14%), specificity (+ 20.35%), and accuracy (+ 17.59) to determine the 1-day fertility window was significantly higher using cervical mucus impedance compared to BBT.

**Conclusions:**

BBT is considered unreliable for evaluating ovulatory function. Cervical mucus electrical impedance offers a novel measure of electrolyte changes associated with hormone levels. We report that pre-ovulatory electrical impedance patterns demonstrated higher sensitivity, specificity, and accuracy for determining the fertility window when compared to BBT. These findings suggest that changes in electrical impedance may provide an accurate method for predicting ovulation and for measuring ovulatory function.

**Supplementary Information:**

The online version contains supplementary material available at 10.1186/s40834-024-00276-w.

## Background

Serum hormone measurements and transvaginal ultrasound examinations are considered the standard for detection of ovulation but, remain cost prohibitive and inconvenient [1, 2]. Due to this, natural family planning techniques for determining the 6-day fertility window including, calendar tracking [3–5], basal body temperature (BBT) [5, 6], and monitoring of cervical fluid changes from estrogen perturbations [7, 8] are widely used. While technically easier and accessible, natural family planning techniques are far less accurate in detecting and predicting the fertility window [1, 5, 6, 9]. These are also inherently subject to issues with user education, compliance, and bias due to various factors. Calendar tracking, including phone applications, often do not account for natural cycle variation [3, 4]. Measurements of BBT are confounded by environmental effects, lack consistent modalities of measurement, and studies, including data reported herein, have shown limited prospective predictability [3, 5, 10]. Cervical mucus evaluation is easily conducted at home but, subjective and generally unreliable [1, 6]. Urine-based test strips to measure luteinizing hormone (LH) offer an improved quantitative metric and are commonly used to detect the spikes in LH that occur 24–36 h prior to ovulation. Studies have found LH measured in urine strongly associates with ovulation, as determined by transvaginal ultrasound [11–13]. However, the LH surge occurs only ∼ 20-hrs prior to ovulation, leaving limited time between detection and potential intercourse before the fertility window closes. Any delay in testing may cause the user to miss her fertile window for that cycle [1, 3, 6]. While these methods offer options for women during family planning, a need exists for more practical, reliable, economical, and predictive tools to determine the fertility window.

A novel alternative predictive measurement to confirm the fertility window is monitoring of cervical mucus electrolyte trends using electrical impedance spectroscopy. Changes to cervical mucus during pregnancy have been observed as early as the nineteenth century [14–17]. Studies from the 1960’s have reported differences in cervical mucus conductivity during pregnancy [14–17] due to electrolyte changes during the pregnancy cycle. The dynamic changes during pregnancy cycle phases with various electrolyte signatures such as sodium, calcium, and potassium in serum has previously been reported, however, this requires blood collection similar to hormone measurements in the clinic setting [18]. Interestingly, it is also known that electrolyte measurements from cervical mucus are associated with hormone level changes during the menstrual cycle.

The purpose of this study was to compare the effectiveness of electrical impedance from cervical mucus and basal body temperature to determine the fertility window. Using a novel at home device, we demonstrate that a rapid and reliable measurement of electrical impedance from cervical mucus, based on hormone level associated electrolyte changes, offers an inexpensive approach for defining the fertility window. We report electrical impedance patterns measured at pre-ovulatory stages demonstrated higher sensitivity, specificity, and accuracy for detecting the fertility window compared to BBT. At home devices are poised to offer longitudinal tracking of these physiological parameters. With personalized data the user can be empowered with improved predictability of fertility and knowledge of reproductive health.

## Methods

### Subjects

This study was carried out at the University Center for Health Sciences, a teaching hospital in Guadalajara Mexico. The study was approved by the Research Committee of Technical Industrial Teaching Center (PI-07-1820). Subjects were informed of the study and selected for screening during routine visits. After screening for eligibility, participants were consented by clinical staff. Subject numbers were assigned sequentially as each woman entered the study. Fourteen [14] women of reproductive age provided written consent for all procedures and were enrolled in this study. All women had a complete gynecological-obstetric history with regularly occurring pap smears. Exclusion criteria included use of hormonal contraceptives and use of including vaginal lubricants, creams, and ointments.

### Study design

All patients participated for three menstrual cycles. Once menstrual bleeding ceased, patients recorded cervical fluid impedance measured via the K-1 Kegg Tracker™ (Lady Technologies, Inc.) and BBT, daily. Patients also used ClearBlue® LH + test strips (SPD, Swiss Precision Diagnostics GmbH, Geneve, Switzerland) until a positive result was obtained. Impedance was measured during the same two-minute interval each day while in the dorsal position, and oral temperature was recorded at the same time as impedance measurement. Data was recorded in the Kegg Tracker™ application program. Impedance was measured in ohms and BBT in °C. All patients were trained on measurement procedures and use of the application prior to start of the study. A representative example of the data obtained for each patient at each cycle can be seen in Fig. 1.

### Clinical confirmation of ovulation

To confirm ovulation, participants received a vaginal ultrasound (SonoAceR3, Samsung Health Care, Seoul, South Korea) and venous blood draw to measure hormone levels within 5 days that a LH + result was obtained. Clinical parameters were conducted by trained nurse practitioners. Ultrasound images were evaluated for follicle number and size in both ovaries. Hormonal levels of LH, follicle stimulating hormone, estradiol, prolactin, and total testosterone were measured using Immunoquimioluminescence following the manufacturer instructions (Vitros 5600, Ortho Inc, California, USA). Physicians, specialized in obstetrics and gynecologists, evaluated all ultrasound images and hormone levels to determine the ovulation date of each cycle for comparison to Kegg™ and BBT values. An additional venous blood draw was conducted 5–7 days post-ovulation to verify normal cycle progression.

### Data analysis and statistics

All statistics was conducted in GraphPad Prism 9.0.0 (San Diego, California, USA), with significance set to *p* < 0.05 for all comparisons. All data was tested for normality using the Shapiro-Wilk test. Kegg™ cervical fluid impedance and BBT were compared between cycle phases using a pairwise Friedman’s test with Dunn’s multiple comparisons tests. Kegg™ cervical fluid impedance and BBT raw data and rate of change values were compared over time using a repeated measured mixed effects model and Tukey’s multiple comparisons. A non-parametric test could not be performed as values were not present for all cycle data evaluated due to patients having different cycle lengths. The relationship between Kegg™ cervical fluid impedance and BBT was conducted using a Pearson’s correlation.

Sensitivity, specificity, and accuracy were calculated by comparing the lowest Kegg™ cervical fluid impedance or BBT value to the determined ovulation date. Sensitivity was defined as the ratio of true positives to true positives and false negatives. Specificity was defined as the ratio of true negative to true negative and false positives. Accuracy was the ratio of the summation of true positive and true negatives to total values evaluated (sum of true positive, true negative, false positive, and false negative values). Sensitivity, specificity, and accuracy were calculated for both a 1-day and 3-day window to determine the ability of using Kegg™ cervical fluid impedance or BBT to predicate ovulation.

## Results

***Patient Demographics***: During the study, two participants were excluded by hormonal disturbance in the second cycle and one for pregnancy. Therefore, data from eleven patients [11] was included in the final analysis. For the final eleven patients, the average age of participants was 30.63 years (range: 21–43 years old), and the median number of children was 1 child (range: 0–2). Two patients self-reported to not be sexually active, while 9 patients self-reported to be sexually active. The average cycle length was 27.93 days (standard deviation of 3.13 days) with the average menstrual length lasting 5.83 days (standard deviation of 1.32 days). The median ovulation day was 13. Complete data sets including basal body temperature and Kegg™ data for all phases of the cycle were recorded in 12 cycles from 9 patients.

**Kegg™*Cervical Fluid Impedance to Determine Cycle Phase***: To determine the utility of using Kegg™ cervical fluid impedance or BBT for determination of cycle phase, cycles values were averaged at each phase of the cycle for each patient and compared. Significant differences were detected in Kegg™ cervical fluid impedance between the follicular phase and ovulation day (*p* = 0.0007) and the luteal phase and day of ovulation (*p* = 0.0007) (Fig. 2A**)**. When individual days were evaluated relative the ovulation day, significant changes in Kegg™ cervical fluid impedance were observed between day − 7 and − 2 of the cycle **(**Fig. 2B**)**. Notably, a trending decrease in Kegg™ cervical fluid impedance was seen during the follicular phase, with significant differences pre-ovulation suggesting Kegg™ cervical fluid impedance could be used to predict ovulation. In contrast, no significant differences in BBT were detected between the follicular phase and ovulation day (*p* > 0.9999) and luteal phase and day of ovulation (*p* = 0.3403) (Fig. 2C**)**. When individual days were evaluated, significant differences in BBT were seen between − 1/-2 and day + 11 **(**Fig. 2D**)**. This suggests that while BBT can be used to detect changes over the cycle period, that changes occur post-ovulation and therefore may not be able to assist to predict ovulation.

***Kegg™ Cervical Fluid Impedance Rate of Change to Predicate Ovulation***: To determine the utility of using Kegg™ cervical fluid impedance to predicate ovulation, the rate of change of Kegg™ cervical fluid impedance or BBT was determined for a -1, -3, -7, + 1, +3, or + 7 day window. Additionally, all follicular (>-7) or luteal ( > + 7) values were considered. For Kegg™ cervical fluid impedance, significant differences were seen between the rate of change for the entire follicular phase (>-7) and all post-ovulation rates (+ 1, + 3, +7, and > + 7) **(**Fig. 3A**)**. This data suggests that changes seen in the data over time can distinguish between the ovulation date. When subjectively evaluating the rate of change data, a phasic characteristic is observed, where the rate is negative, then becomes positive during the fertility window (-1). In contrast, no significant differences in the rate of change for BBT were detected (*p* > 0.05) **(**Fig. 3B**)**.

***Kegg™ Cervical Fluid Impedance Superiority to BBT to Predict Ovulation***: Sensitivity, specificity, and accuracy for Kegg™ cervical fluid impedance and BBT were calculated to determine the utility of these measures to predict the ovulation day based on clinical parameters (ultrasound and hormone blood panel). Notably, for both a -1 and − 3 day window Kegg™ cervical fluid impedance had higher sensitivity, specificity, and accuracy values relative to BBT **(Supplementary Table 1)**. This suggests that Kegg™ cervical fluid impedance is a superior value to predict ovulation relative to BBT.

***Relationship Between Kegg™ Cervical Fluid Impedance and BBT***: Interestingly, no correlation between Kegg™ cervical fluid impedance and BBT was detected for all phases **(**Fig. 4A**)**, or individual phases of the cycle including the ovulatory (ovulation day) **(**Fig. 4B**)**, follicular phase **(**Fig. 4C**)**, or luteal phase **(**Fig. 4D**)**. While the exact mechanism of how cervical fluid impedance changes throughout the cycle is unknown, no correlation between Kegg™ cervical fluid impedance and BBT may suggest that Kegg™ cervical fluid impedance is a novel avenue to study to evaluate cycle changes associated with ovulation, but also reproductive health.

## Discussion

The average menstrual cycle contains approximately six fertile days that are often referred to as the “fertility window” and include the five days prior to and with the day of ovulation. The day prior to ovulation is typically considered the most fertile day. The fertility window closes shortly after ovulation since the egg loses its ability to be fertilized not many hours after ovulation. Our results demonstrate that cervical fluid impedance, as measured by the Kegg™ device, offers improved rapid determination of the most fertile day when compared to alternative methods.

It is known that there is significant cycle to cycle variation within and among women in the day of ovulation and occurrence of the fertility window [19–21]. Serial serum hormone measurements and transvaginal ultrasound are measures commonly used to predict the fertility window but are inconvenient and require office visits. Other methods such as luteinizing hormone (LH) urine strips and basal body temperature (BBT) are commonly used as an alternative but are generally unreliable.

Infertility is a disease recognized by various organizations including the World Health Organization and American Society for Reproductive Medicine [22]. According to the American Society for Reproductive Medicine, infertility is generally defined as a lack of ability to conceive after 12 months of unprotected sex due to impairment by the patient or partner [21]. The position of the American Society for Reproductive Medicine (ASRM) is to evaluate and/or treat infertility at ≥ 12 months in women under 35 years of age and ≥ 6 months in women above 35 years of age [21, 22]. Evaluation includes a review of the patient’s medical history and for existing known pathologies effecting fertility [21, 23]. The ASRM recommends initial diagnostic methods for infertility to include assessments for ovulatory function given it accounts for approximately 40% of infertility cases in women [24]. For remaining cases, infertility may be caused by lack of ovarian reserve, abnormalities of the cervix, fallopian tubes, or uterus in addition to conditions of the partner all of which can be subsequently evaluated and treated through various methods reviewed and recommended elsewhere [21].

In clinical practice, ovulatory function is routinely determined across multiple cycles via measurement of luteal progesterone, use of ovulation prediction kits, or through transvaginal ultrasound which is the most commonly used technique by reproductive endocrinologists and infertility specialists [2, 21]. Measurements of basal body temperature (BBT) and cervical fluid viscosity have historically been used as predictors of ovulation. However, results from various clinical studies have demonstrated that these methods are significantly unreliable and subjective especially in women with conditions such as polycystic ovary syndrome (PCOS) [1, 5, 6, 9]. Serum luteal progesterone (PG) may provide an accurate predictor of recent ovulation but requires collection at specific times during the fertility window at 1 week prior to menses [21, 25]. PG concentrations overtly surge during the luteal phase thus a single luteal PG measurement of > 3 ng/ml is usually sufficient to indicate recent ovulation although serial measurements are more conclusive and serum PG lacks the ability to determine quality of the luteal phase [21, 25]. Ovulation predictor kits to measure urine LH levels offer an additional tool to predict ovulation in more practical at-home settings. Urine LH levels do seem to be commensurate with increased serum levels but time of day for collection (midday or evening preferred) and accuracy variance among kits must be considered [21, 26]. Urine LH tests are also limited considering its an indirect measure [27] and the fact that LH spikes occur later in the fertility window, usually within 1–2 days of ovulation, which adds some difficulty in conception planning. False positive OPK spikes are also often noted. Transvaginal ultrasonography is regarded as the “gold standard” for evaluation of ovulatory function. This method allows for not only assessment of developing follicles and evidence for luteinization/ovulation, but additionally allows for the evaluation of ovarian reserve and signatures of uterine or cervical pathology [21]. Transvaginal ultrasonography is however somewhat limited by the need to take measurements in the predicted luteal phase for evidence of ovulation [21].

A central problem in women with infertility is the reliable ability to accurately detect and predict the fertility window. Current methods as described above lack reliability or require office visits and related costs in specific cycle windows for best results. Furthermore, for women with hormonal imbalances that effect fertility such as hyperprolactinemia, oligomenorrhea, amenorrhea, or PCOS, current ovulation detection methods measuring urine LH or serum PG are especially lacking as they can produce false positives or false negatives [21]. Here, we report the increased accuracy, sensitivity, and specificity of cervical fluid electrical impedance patterns in predicting the fertility window versus serial BBT measurements using an at-home device capable of rapid detection of electrolyte trends via electrical impedance spectroscopy. Changes in the cervical mucus throughout pregnancy have long been observed clinically [1, 6], but have not been objectively evaluated as a predictive ovulation tool, or compared to historical patient determined methods such as BBT.

## Conclusions

Current methods to predict the fertility window are limited by cost, need for an in-office evaluation, and lack of accuracy and reliability. The determination of the ovulatory window with alternative yet reliable at-home assessments could provide assurance and predictability for natural family planning and for women with diagnosed infertility who are trying to conceive. In this study we demonstrate a rapid and reliable means to measure cervical mucus electrical impedance based on electrolyte changes in cervical mucous previously known to be associated with circulating hormone levels indicative of ovulation. Electrical impedance patterns measured at pre-ovulatory stages were determined to have higher sensitivity, specificity, and accuracy for the detection of the fertility window compared to BBT when confirmed by trans-vaginal US. While applications in women with irregular cycles has yet to be evaluated, these methods may add more sensitive predictive ability in detecting the fertile window in women with infertility but additionally as part of routine family planning.

### Electronic supplementary material

Below is the link to the electronic supplementary material.

Supplementary Material 1

## Data Availability

The datasets used and/or analysed during the current study are available from the corresponding author on reasonable request.

## Abbreviations

BBT: Basal Body Temperature
OPK: Ovulation Predictor Kit
LH: Luteinizing Hormone
ASRM: American Society for Reproductive Medicine
PCOS: Polycystic Ovary Syndrome
PG: Luteal Progesterone
