# Supplementary material for: The effectiveness of cervical mucus electrical impedance compared to basal body temperature to determine fertility window
Source: Contracept Reprod Med. 2024 May 6;9:20. doi: 10.1186/s40834-024-00276-w (PMC11071211; doi:10.1186/s40834-024-00276-w)
Supplement: Supplementary file 1 — Supplementary Material 1 [file 40834_2024_276_MOESM1_ESM.docx]

**Supplemental Table 1.** Summary of sensitivity and specificity values in determining the ovulatory phase relative to follicular phase for Cervical Fluid Impedance relative to an accepted form of ovulatory predictor: basal body temperature. Averaged percentages to demonstrate trends.

**
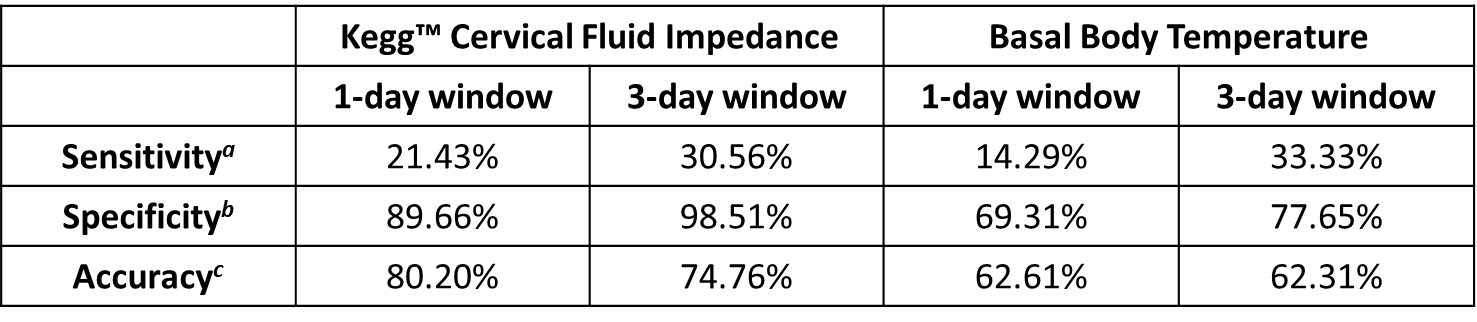
**

*^a^*Sensitivity (True Positive Rate) refers to the probability of a positive test, conditioned on truly being positive.

*^b^*Specificity (True Negative Rate) refers to the probability of a negative test, conditioned on truly being negative.

*^c^*Accuracy refers to the proportion of true results, either true positive or false negative.
